# *In vitro *identification and *in silico *utilization of interspecies sequence similarities using GeneChip^® ^technology

**DOI:** 10.1186/1471-2164-6-62

**Published:** 2005-05-04

**Authors:** Dmitry N Grigoryev, Shwu-Fan Ma, Brett A Simon, Rafael A Irizarry, Shui Q Ye, Joe GN Garcia

**Affiliations:** 1 Center for Translational Respiratory Medicine, Division of Pulmonary and Critical Care Medicine, Johns Hopkins University, 5501 Hopkins Bayview Circle, JHAAC/4A.24, Baltimore, MD 21224, USA; 2 Department of Anesthesiology and Critical Care Medicine, Johns Hopkins University, 600 North Wolfe Street, Tower 711, Baltimore, MD 21287, USA; 3 Department of Biostatistics, Johns Hopkins University,615 N. Wolfe Street, E3035, Baltimore, MD 21205, USA

## Abstract

**Background:**

Genomic approaches in large animal models (canine, ovine etc) are challenging due to insufficient genomic information for these species and the lack of availability of corresponding microarray platforms. To address this problem, we speculated that conserved interspecies genetic sequences can be experimentally detected by cross-species hybridization. The Affymetrix platform probe redundancy offers flexibility in selecting individual probes with high sequence similarities between related species for gene expression analysis.

**Results:**

Gene expression profiles of 40 canine samples were generated using the human HG-U133A GeneChip (U133A). Due to interspecies genetic differences, only 14 ± 2% of canine transcripts were detected by U133A probe sets whereas profiling of 40 human samples detected 49 ± 6% of human transcripts. However, when these probe sets were deconstructed into individual probes and examined performance of each probe, we found that 47% of human probes were able to find their targets in canine tissues and generate a detectable hybridization signal. Therefore, we restricted gene expression analysis to these probes and observed the 60% increase in the number of identified canine transcripts. These results were validated by comparison of transcripts identified by our restricted analysis of cross-species hybridization with transcripts identified by hybridization of total lung canine mRNA to new Affymetrix Canine GeneChip^®^.

**Conclusion:**

The experimental identification and restriction of gene expression analysis to probes with detectable hybridization signal drastically increases transcript detection of canine-human hybridization suggesting the possibility of broad utilization of cross-hybridizations of related species using GeneChip technology.

## Background

Genome-wide analyses of multiple organisms in a variety of experimental biological systems are powerful tools employed in biomedical research. However, microarray platforms for numerous species, particularly large mammals, have yet to be fully developed. Large mammals, often preferred species for modeling of various pathophysiological conditions and environmental responses, demonstrate better concordance to humans with regard to toxicological effects compared to small animal models (rodents) [1,2]. Further, large animal models are indispensable in disease modeling that requires longer live span [3], in organ transplant research [4], and in studies where multiple samples are required to be collected [5]. For organ- and system-specific applications, such as the respiratory system, canine and ovine lungs are physiologically and hemodynamically more closely related to human lungs than rodent lungs and allow regional evaluation of air, blood, and exudate distribution. Thus, canine [6,7] and ovine [8,9] models are the most commonly utilized in pathophysiological modeling of the respiratory system.

Despite these obvious advantages of the large mammal model, the lack of large mammal genomic sequence data presents significant limitation for genome-wide analysis of gene expression profiles by microarray techniques. To overcome this limitation, cross-species RNA hybridization has been utilized as one potential solution [10-13]. This approach is based on the notion that evolutionarily-conserved genetic sequences between mammals will be efficiently detected during cross-species hybridization. Despite a paucity of literature focused on the use of Affymetrix GeneChip oligonucleotide array for cross-species hybridizations [14,15], we speculated that this array platform could potentially serve as a powerful tool for cross-species genome analysis. GeneChips carry 11–16 oligonucleotide probes per target gene, increasing the opportunity to potentially identify genes with stretches of high homology to specific probes. To test this hypothesis, we cross-hybridized total RNA obtained from canine lung tissues to the human HG-U133A GeneChip. Analysis of generated expression profiles using Affymetrix MicroArray Suite (MAS 5.0) showed that approximately 14% of canine mRNA hybridizes to the human probe sets and generates hybridization signal similar to that of human mRNA. Analysis of available canine orthologues corresponding to these well performing human probes revealed high sequence similarities between human and canine counterparts. Based on these findings we speculated that even canine genes with only partial sequence similarities to their human counterparts can be identified by MAS 5.0 if the analysis is restricted to probes with high sequence similarity to their canine counterparts. Antipova *et al*. recently reported that utilization of a single well performing Affymetrix probe pair can be sufficient for detection of its corresponding target [16], further supporting our hypothesis.

A straight forward approach to test this hypothesis would be to align the target sequences from the human HG_U133A GeneChip to the known canine sequences using Basic Local Alignment Search Tool (BLAST), followed by identification of human probes with high sequence similarity to their canine counterparts. Unfortunately, due to limited canine genomic information, only 3% of human target sequences from the HG_U133A GeneChip have been matched to known canine orthologues, which renders this approach inadequate. However, we speculated that since canine orthologues with high sequence similarities to human probes generate detectable hybridization signals, the reverse should be also true: the detectable hybridization signal should reflect high sequence similarity. Therefore, we examined individual probes instead of complete probe sets. Probes with detectable hybridization signals were considered adequate representatives of their corresponding canine orthologues and were thus employed for final signal intensity calculations. The MAS 5.0 analysis when restricted to probes with detectable hybridization signals, demonstrated a 4-fold increase in canine transcript detection as compared to unrestricted analysis. Our results strongly suggest that identification of probes with detectable cross-species hybridization signal and subsequent restriction of gene expression analysis to these probes can be applied to multiple related species for which gene arrays are not commercially available.

## Results

**Figure 1:**
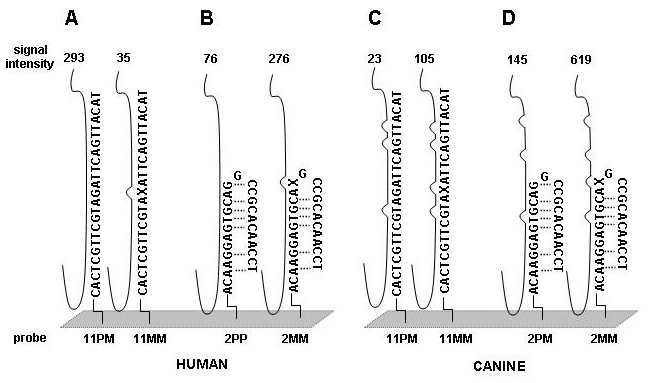
**Hybridization pattern of human (panels A and B) and canine (panels C and D) mRNAs to 2^nd ^and 11^th ^probe pairs of 203133_at Affymetrix probe set**. The 203133_at probe set contains 11 probe pairs of which 2^nd ^and 11^th ^represented by anchored oligonucleotide sequences. Corresponding probe pairs identified at the bottom and comprised of perfect match probe (PM) and mismatch probe (MM). The purposely mutated 13^th ^nucleotide in MM sequences is shown as "X". The target mRNAs represented by solid lines aligned to oligonucleotide sequences and mismatched regions depicted as humps. Numbers on the top of each target mRNA represent intensity values generated by MAS 5.0 (Figure 2 A and B). Dotted lines depict nucleotide bond involved in formation of predicted hairpin structure of the 2^nd ^probe of the 203133_at probe set.

**Figure 2:**
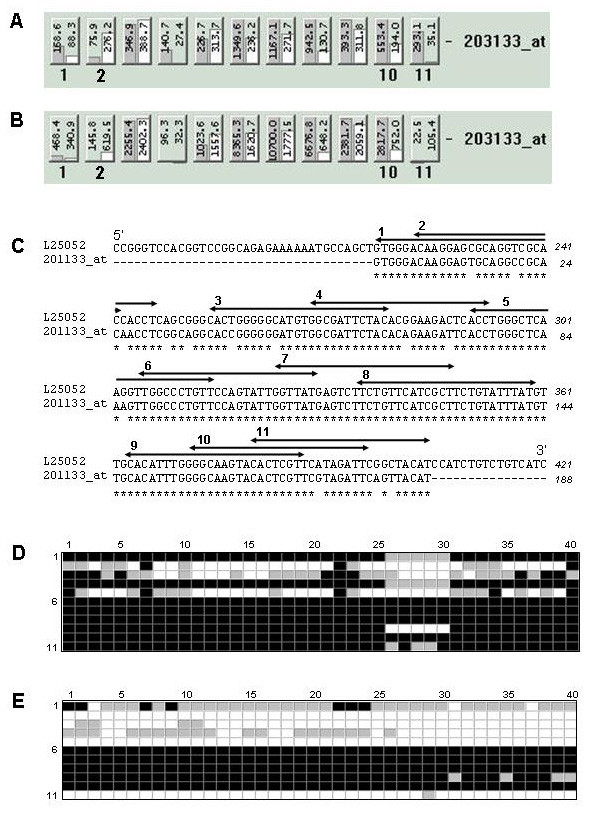
**Hybridization patterns of human and canine SEC61B gene to Affymetrix 203133_at probe set and alignment of 203133_at probe pairs to canine SEC61B gene sequence**. Panels A and B represent screen shots of MAS 5.0 generated intensities for "perfect match" probes (grey columns) and "mismatched' (open columns), respectively. Panel C depicts CLUSTALW generated alignment of canine SEC61B gene (accession number L25052) and 11 "perfect match" probes of Affymetrix 203133_at probe set. Star signs represent matched base pairs and double-headed arrows show locations of 203133_at probes (the corresponding probe number attached at the 5' end of probes). Panel D represent human SEC61B gene hybridization patterns throughout 40 GeneChip arrays. The order numbers of 203133_at probes are shown on the left. GeneChips were grouped by tested tissues (8 tissue groups with 5 chips per group) and are labeled on the top of each profile. Probe pairs were divided into three groups: poorly performing (open blocks, (*PM *- *MM*) ≤ 20), moderately performing (grey blocks, 20 < (*PM *- *MM*) ≤ 200), and well performing (solid blocks, (*PM *- *MM*) > 200). Panel E represent canine SEC61B gene hybridization patterns throughout 40 GeneChip arrays.

The hybridization pattern of the protein translocation complex beta gene involved in cell maintenance (SEC61B, Gene Ontology #8151) is assumed to be constantly expressed in the majority of tissues and was selected for demonstration of the probe level analysis. This analysis was conducted using signal intensity values generated by hybridization of human and canine SEC61B mRNAs to HG-U133A GeneChip. The probe level analysis deconstructs the entire Affymetrix probe set into its individual components and allows one to evaluate the performance of each individual probe. Detailed output of human SEC61B hybridization to 203133_at probe set (Figures 1A and 2A) shows that signal intensity value of PM (perfect match or total signal) obtained for 11^th ^probe pair is higher than that of MM (mismatch or background signal). Therefore, correction for the background will produce positive signal intensity value, and this probe pair will contribute to the "Present" (detectable transcript) classification by MAS 5.0. However, cross-species hybridization of canine mRNA to the same probe pair does not produce sufficient signal intensity value (Figures 1C and 2B), possibly due to differences in target sequence between canine and human. This will, therefore, shift the overall probe set classification towards "Absent" (non-detectable transcript) classification. At the same time, there are probes that failed to detect both human and canine SEC61B. This could be due to either secondary structure formation (Figure 1B and 1D) [17] or the presence of sequences in the hybridizing mRNA mixture that possess homology to the 11^th ^MM probe, thus raising the background signal over the specific signal. The pattern of SEC61B hybridization shows that, among 11 probe pairs of the 203133_at probe set, 3 probes (#2, #3, and #5) failed to detect its target in human tissues but the detectable hybridization of the remaining 8 probes prevailed (Figure 2A), resulting in classification of the SEC61B transcript by MAS 5.0 as "Present". In contrast, a reduction in the number of probes that were able to detect canine SEC61B down to only 6 probes (#1, ##6–10, Figure 2B) caused MAS 5.0 to classify this gene as "Absent". As shown in Figure 2C, the failure of the 5 non-hybridizing canine SEC61B probes was mainly attributed to differences in gene sequences between these two species.

This observation held true for most of the evaluated targets, leading us to speculation that among the 11 human probes of a given probe set there will be probes with high sequence similarity to its canine target and that identification and utilization of these probes will increase sensitivity of cross-species hybridization. Moreover, when we evaluated performance of all 9,871,960 HG-U133A probes from 40 cross-species hybridizations, we found that almost half of these probes (4,688,566) generated a detectable hybridization signal (PM-MM>20). Therefore, we speculated that excellent and poorly performing probes should be equally distributed in the probe sets as well, and that masking of the poorly performing half will increase transcript detecting ability of these modified probe sets. The test run of MAS 5.0 analysis using masking of the 5 poorly performing probes of 203133_at probe set converted the initial "Absent" call for canine SEC61B gene to a "Present" call and will formulate the basis for further investigation. Since the canine genome is not yet completed, we were unable to select probes with high canine/human sequence similarity solely based on gene sequence information. Instead, we evaluated the overall performance of each individual probe based on their hybridization signal. We speculated that sequences of human probes with a reasonable cross-species hybridization signal (PM-MM>20) most likely possess a high similarity to their canine targets.

**Figure 3:**
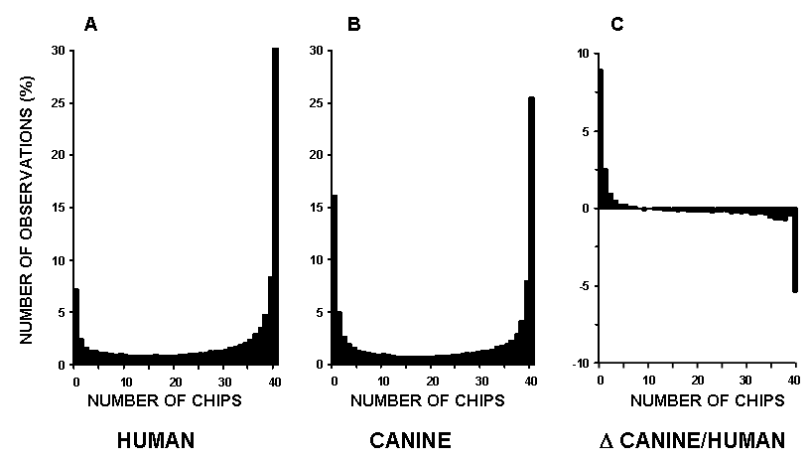
**Histogram of probe performance during human and canine samples hybridizations to HG-U133A**. The number of hybridizations that generated detectable signal for individual probe throughout 40 GeneChips was computed and histograms for 246799 analyzed probes were generated for human (panel A) and canine (panel B) samples. Panel C represents the net effect of canine sample on probe performance.

**Figure 4:**
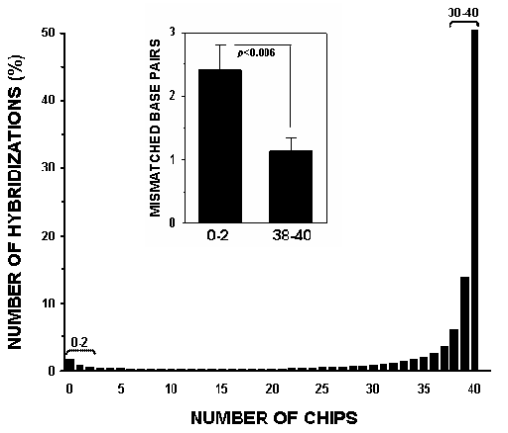
**Effects of human/canine genomic differences on probe performance and correlation of probe performance with sequence similarity**. Only well performed during human/human hybridization probes were employed for analysis of the effects of genetic sequence differences between human and canine. Two pools of transcripts were probes with non-detectable hybridization signal in 95% cases (generated detectable signal during 0–2 hybridizations) and probes that generated detectable signal in 95% cases (38–40 hybridizations). Randomly selected probes from each pool were aligned to their canine counterparts and number of mismatched base pairs was identified. The difference in detected mismatches between these probe pools was evaluated using Mann-Whitney test with *p *< 0.05 used as a significance cut-off (insert).

The probe level analysis was performed for 40 expression profiles generated by hybridization of 8 different human tissues and 40 expression profiles generated by hybridization of canine lung tissues to the human HG-U133A GeneChip. The resulting hybridization signal pattern of the 203133_at probe set for both species is presented in Figure 2D and 2E. As expected, differences in the canine SEC61B mRNA sequence region corresponding to the 11^th ^probe (Figure 2C) did not allow canine target detection in 39 out of 40 hybridizations (Figure 2E). These data suggest that once a probe poorly hybridizes to its target in several initial experiments, it will most likely fail to detect its target in the substantial number of consequent hybridizations. To investigate whether this pattern persists throughout whole gene array, we analyzed hybridization patterns of all 246799 probes of the HG-U133A GeneChip. The resulting histogram for the same species hybridization (Figure 3A) demonstrates that a large fraction (~44% cases) of probes performed well during 38 or more hybridizations (95%). This trend was also true for poorly performing probes, where a considerable fraction (~11%) probes failed to detect their target during 38 or more hybridizations. In canine/human hybridization the number of poorly performing probes was higher (24%) while fraction of well performing probes lowered to 38% (Figure 3B). The analysis of the overall net effect of canine/human sequence differences on the hybridization pattern was conducted by correction for poorly performing probes during human samples hybridization. This revealed a 7% decrease in well performing and 13% increase in poorly performing probe fractions (Figure 3C). For detailed evaluation of direct effects of interspecies sequential differences on the cross-species hybridization, we selected probe sets where all probes generated detectable hybridization signal during human sample hybridization. Interestingly, among these probes, the fraction of probes that detected their corresponding canine targets during 38 or more cross-species hybridization was surprisingly high (~72%), with a very low (~3%) fraction of probes that failed to detect their targets in canine tissues (Figure 4). To evaluate correlation between performance of an individual probe and its alignment to the corresponding canine target, the number of mismatches were identified and counted for 20 randomly selected canine genes. This analysis demonstrated that, on average, probes in both excellent and poorly performing fractions had 1 and 2.5 mismatched bases, respectively, with the mismatch rate significantly lower in excellent performing probes (Figure 4, insert). The presence of a mismatch in probes with detectable hybridization signal was not unexpected and correlates with previously reported observation that relatively long (≥ 16 bp) stretch of the perfect match could produce stable hybridization and signal generation [15].

**Figure 5:**
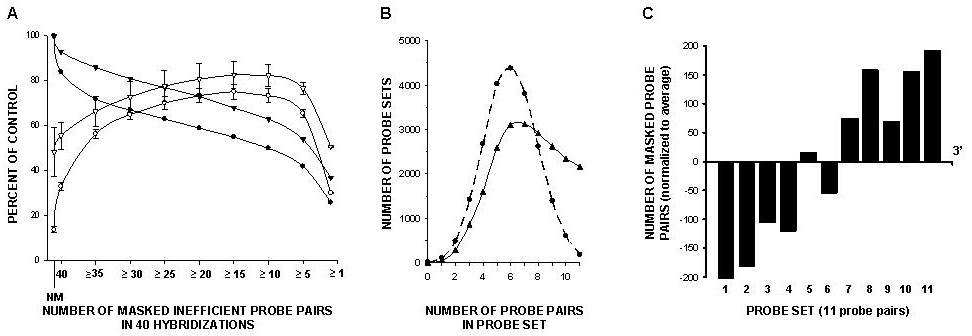
**Effects of masking of poorly performed probes during human and canine hybridizations to HG-U133A on "Present" calls, average probe set size and composition**. **Panel A**. MAS 5.0 was applied to human (open triangles) and canine (open circles) hybridization profiles without masking (NM) and with masking of probes that poorly performed in 40, ≥35, ≥30, etc. hybridizations (*x *axis) and resulting numbers of total "Present" calls generated for each array were averaged and expressed in percentage values (*y *axis). Average sizes of human (solid triangles) and canine (solid circles) probe sets were computed for corresponding masking conditions and expressed in percentage values (*y *axis). **Panel B**. Distribution of probe set sizes after masking probes that failed to generate detectable hybridization signal during 15 or more hybridizations (~40%) of human (triangles) and canine (circles) samples. **Panel C**. Distribution of masked probes according to location on the target sequence.

The criterion for masking of a particular probe was based on the performance of a given probe throughout 40 GeneChips. We found that masking of probes which poorly performed (PM-MM≤20) in 15 or more hybridizations (~40% of all hybridizations) produced the highest number of "Present" calls (Figure 5A) therefore this percentage was selected as a cut off value for masking. We speculated that probes that failed to detect its target in more than 40% of hybridizations lack homology to their canine counterparts at least in part due to sequence divergence. To test this hypothesis we conducted a sequence analysis of randomly selected samples and find that out of the 42 tested probes 27 probes aligned against canine sequence with one or more mismatches and 22 of these probes (approximately 82%) were masked by our procedure.

We also analyzed the effects of our procedure on the probe set size (Figure 5B). The main fractions of probe sets adjusted to human (77% of all probe sets) or canine (81% of all probe sets) hybridizations are represented by 5–10 and 4–8 probe pairs, respectively. Our masking procedure left 2172 (10%) probe-sets unaltered and completely eliminated 7 (0.03%) probe sets based on the human hybridization pattern. Analysis of canine hybridization identified 184 (0.8%) highly homologous canine targets, which hybridize to the entire corresponding human probe sets, and eliminated 11 probe-sets (0.05%) where none of the probes recognizes its target in canine tissues.

We next examined relative location of poorly performing human probes on the canine genes. It has been reported previously that human/canine homology is prone to be higher within the coding region [14]. Our findings are consistent with this report and show that our approach masked more probes towards the 3' untranslated region of target sequences (Figure 5C).

**Figure 6:**
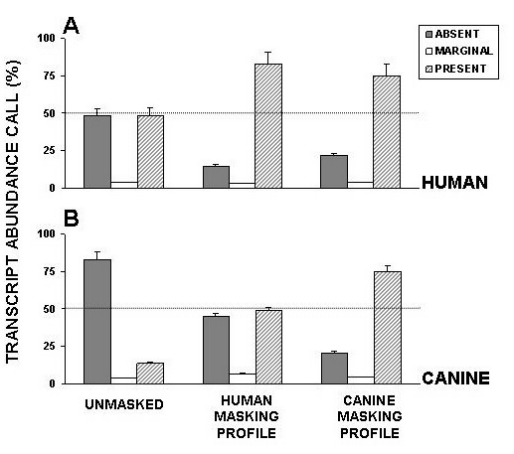
**Comparison of transcript-detecting ability before and after masking procedure**. Solid bars represent percentage values of "Absent" transcript abundance calls. Open and grey bars represent "Marginal" and "Present" transcript abundance calls, respectively.

The effect of our masking procedure was also evaluated for transcript abundance detection. The combined (40 GeneChips) output of regular MAS 5.0 analysis was compared with outputs produced using masking files generated for human and canine hybridizations (Figure 6). In human hybridization, our algorithm reduced "Absent" calls by 3 fold and increased "Present" calls by more than 1.5 fold. The application of the masking file generated for canine hybridization had a more modest effect than human-specific masking. The transcript detection during the hybridization of canine mRNA to the human HG-U133A chip was much less sensitive (14%) than that of hybridization of human mRNA (48%). Our canine-specific masking recovered approximately 60% (Figure 6) of transcripts which, otherwise, were called "Absent" by MAS 5.0. The anticipated increase in transcript detection by application of human-specific masking is attributed to the masking of imperfectly designed probes. As shown in Figure 1, the 11^th ^probe of the 203133_at probe set failed to detect its target in both canine and human tissues. Therefore, masking of such a probe will improve transcript detection in both canine and human sample analyses. Similarly, the canine-specific masking of human hybridization also increased the number of detectable transcripts as compared to non-masking results (Figure 6A).

**Figure 7:**
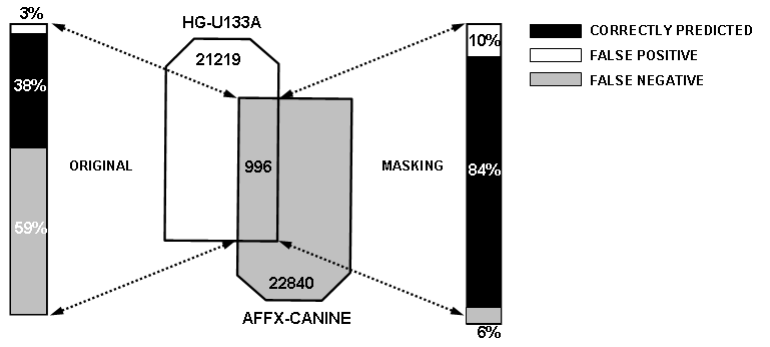
**Comparison of transcripts detected by cross- and same-species hybridization**. The common orthologues presented on HG-U133A and Affymetrix canine GeneChips were identified (996 orthologous pairs) using MegaBLAST. Transcripts identified by hybridization of canine sample to Affymetrix canine GeneChips were compared with those identified during hybridization of canine sample to human HG-133A GeneChips with and without application of masking procedure. Correct (solid bars), false positive (open bars) and false negative (grey bars) signal intensity calls were identified and expressed in percentage values.

Finally, we compared transcripts detected by our masking procedure with those identified by hybridization of canine lung tissues to the newly developed Affymetrix canine GeneChip. We have identified 996 orthologous transcripts common to both HG-U133A and canine GeneChips and compared their detection in both arrays using "Present"/"Absent" classifications by MAS 5.0 or GCOS 1.1, respectively. This analysis showed that our masking approach increased the fraction of correctly identified transcripts by 46% and decreased the false negative fraction by 53% as compared to unmasked transcript detection (Figure 7).

## Discussion

The widespread use of large mammal models in respiratory research, coupled to the limited availability of corresponding gene microarrays, motivated us to investigate the feasibility of utilizing gene arrays designed for related organisms. In this work, we hybridized canine lung tissues to human Affymetrix HG-U133A GeneChip, and demonstrated that oligonucleotide probes designed for identification of human targets were able to generate detectable hybridization signal for canine tissues (Figure 6B). However, the number of identified transcripts was 3–4 times lower than that of the transcripts which were identified during human sample hybridization (Figure 6A). Our analysis indicated that the low sensitivity of the majority of the HG-U133A probe sets to canine transcripts was due to partial genomic differences between human and canine sequences (Figure 2B and 2C). The abundance of probes in Affymetrix probe sets (11–16 probes per set) presented an opportunity for reduction of probe sets to probes that generate detectable cross-species hybridization signal and therefore, improving transcript detecting ability of such modified probe sets. Our probe level analysis allows evaluation and identification of probes that failed to detect their targets in canine tissues. We demonstrated that this poor performance was not random, but rather linked to either imperfect probe design (Figure 1B) or interspecies differences in genomic sequences (Figure 4). Another indirect linkage of probe/target sequence similarity and probe performance was demonstrated by inclination of poorly performing probes toward the 3' end of a target (Figure 5C), which is more prone to diversity [14]. The masking of this poorly performing probes resulted in an increase of "Present" calls in both species (Figure 5A), where the increase in human transcript detection was attributed mainly to masking of imperfectly designed probes. Interestingly, the increase of "Present" calls was more prominent in canine hybridizations when we masked the most poorly performing probes that were not able to detect their targets in more that 30 hybridizations. However, thereafter the difference in number of "Present" calls between canine and human remains constant (Figure 5A). This comparable increase in the amount of "Present" calls is most likely species-independent and attributed to probe set optimizations, which is reflected by changes in probe set sizes. We demonstrated that masking probes that were not detecting their targets in 40% cases generated maximum of "Present" calls in both species (Figure 5A) and speculated that probe pairs that poorly performed in more than 40% of hybridizations lack homology to their canine counterparts. This was confirmed by alignment analysis of these probes to their canine counterparts (Figure 2), which showed that 82% of masked probes produced one or more mismatches with their canine counterparts. The remaining 18% can be attributed to a sequencing error, a single nucleotide polymorphism, or longer than 16 bp stretches of perfect matches that can produce detectable hybridization signal [15]. We also identified probes that were perfectly aligned to their canine counterparts yet perform poorly. These probes represented approximately 7% of tested probe pairs, a value similar to that previously reported [17] and can be attributed to the secondary structure or to the presence of yet unknown sequence that recognizes MM probe as a perfect match and the resulting increase in background signal obscure the hybridization signal.

We demonstrated that modification of probe sets by masking poorly performing probes drastically increased detection of canine transcripts by the human HG-U133A GeneChip (Figure 6B). To validate these results, we compared transcripts identified by our masking approach with those identified by hybridization of canine lung tissues RNA to the Affymetrix canine-specific GeneChip. This comparison demonstrated that modification of probe sets prior to MAS 5.0 analysis drastically increases correlations between transcripts identified by same- and cross-species hybridizations (Figure 7). The slight increase (from 3% to 10%) in false positive transcript fraction introduced by our procedure is a common feature of high density oligonucleotide array analyses [18] and is well compensated by the approximately 10-fold decrease in the false negative transcript fraction from 59% to 6% (Figure 7).

## Conclusion

The studies presented here show that the Affymetrix microarray platform can be successfully used for cross-species hybridization of related organisms. A simplified version of this method (probes that failed to generate detectable signal in all hybridizations were masked) has already been successfully applied to small (7 GeneChips) data sets [19] and produced biologically sensible results. We expect that method presented here will be applicable not only for canine/human hybridization but also for other combinations of cross-species hybridizations. Whenever there is homology between species, there is an opportunity for identification probes with detectable hybridization signal using probe level analysis described here. This method will accelerate genome-wide analysis in experimental models for which species-specific gene arrays are not currently available.

## Methods

### Animal preparation and sample collection

All experimental procedures were approved by the Johns Hopkins University Animal Care and Use Committee. Four mongrel dogs (weight 21.3 ± 1.5 kg) were anesthetized with 25 mk/kg pentobarbital i.v. and instrumented with femoral arterial and venous catheters. Anesthesia was maintained with additional pentobarbital (5 mg/kg iv every hour and when indicated) and muscle relaxation provided by pancuronium (3 mg bolus and 0.5 mg hourly iv). A 39 or 41 French double-lumen endobronchial tube (Mallinkrodt, St. Louis, Missouri) was placed via a tracheostomy and left lung was then mildly injured by repeated lavage with warmed saline. One animal was sacrificed by exsanguination after supplemental pentobarbital (10 mg/kg i.v.) right after lavage, the chest opened, and 10 control tissue samples were taken from 5 corresponding regions in both lungs (apex- dependent and non-dependent, base- non dependent, mid, and dependent). Other 3 animals were ventilated for 5 hours, then sacrificed as described above and 30 samples (10 from each animal) were collected following the control animal pattern. The lung tissue samples were immersed in RNAlater (Ambion, Austin, Texas), snap-frozen and stored at -80°C until processed for RNA isolation. The protocol for cross-species hybridization sample preparation has been described by our group previously [20,21]. Briefly, total RNA was isolated from individual lung samples described above using the Trizol reagent and Qiagen RNeasy columns. Individual cDNAs were prepared from each RNA isolate using reverse transcriptase (GIBCO-BRL SuperScript). Each cDNA was subsequently used as a template to make biotin-labeled cRNA using an *in vitro *transcription reaction (Enzo), resulting in a single cRNA for each lung sample. Each cRNA was hybridized with an individual Affymetrix HG-U133A oligonucleotide array, which was subsequently processed and scanned according to the manufacturer's instructions. All arrays were hybridized in the same batch to avoid variability in hybridization conditions. The sample collection and preparation for the same specie hybridization was adapted from the described above protocol and applied for Affymetrix Canine GeneChip hybridization by McVerry et al [22].

### Human tissues and cell lines sampling and RNA isolation

All human samples and analyses posted on the HopGene server were generated with IRB approval (#B0112210102). Pathologic samples of human myocardium 1 from unused donor heart, 2 from patients with ischemic cardiomyopathy, and 2 from patients with nonischemic cardiomyopathy were collected at time of transplantation. Peripheral blood mononuclear cells were collected form 1 healthy person, 2 smokers, and 2 patients with allergic asthma and separated by Ficoll-hypaque density gradient centrifugation. Airway brushing cells were collected in normal saline form 1 healthy person, 3 smokers, and 1 patient with allergic asthma and separated by centrifugation. Bronchoalveolar lavage (BAL) was performed during bronchoscopy of 2 healthy individuals, 2 smokers, and 2 patients with allergic asthma by instilling 100 ml of normal saline, BAL fluid was aspirated into tubes and cells collected by centrifugation. The muscle tissue was collected from 5 individuals with different stages of obesity. All human tissues were snap-frozen in liquid nitrogen upon collection. These frozen samples (~50 mg from each sample) were directly solubilized in chaotropic solubilization buffer using a Brinkman Polytron tissue disruptor. Larger tissue fragments (>100 mg) were pulverized into frozen powder with a mortar and pestle, pre-chilled to liquid nitrogen temperature, and then the frozen powder was solubilized with the Polytron. RNA was purified using Trizol LS (Life Technologies) and an additional RNA purification step was conducted using the RNAeasy purification kit (Qiagen Inc., Valuencia, CA).

Human smooth muscle cell cultures were treated with ApoC-1 for 10 min (n = 2), 24 h (n = 2) or left intact (n = 1); human pulmonary artery endothelial cells were exposed to hyperoxia for 6 h (n = 1), 24 h (n = 1), 48 h (n = 1), or normoxia (n = 2); IB3-1 bronchial epithelial cells were treated with 1 mM PBA for 24 h (n = 1) or left intact (n = 4). Cells were rinsed twice with ice cold Hank's buffer then scraped into 3ml of ice cold TRIzol (Gibco BRL). Total RNAs was purified using Trizol LS (Life Technologies) and an additional RNA clean-up step was conducted using the RNAeasy purification kit (Qiagen Inc., Valuencia, CA). Approximately 10 μg of purified, total RNA was used for analyses.

### RNA preparation and hybridization

Purified total RNA was reverse transcribed to first-strand cDNA using a hybrid primer consisting of oligo-dT and T7 RNA polymerase promoter sequences. The single-stranded cDNA was then converted to double-stranded cDNA. Complementary DNA corresponding to 5–10 μg of total RNA was used in a cRNA amplification step using T7 RNA polymerase and two biotinylated nucleotide precursors. The resulting biotinylated cRNA was fragmented to a size of approximately 50 bp. Approximately 20–30 μg of cRNA from each tissue was hybridized to corresponding (HG_U133A or Canine) GeneChips (Affymetrix, Santa Clara, CA). The bound cRNA was visualized by binding of streptavidin/phycoerythrin conjugates to the hybridized GeneChip, followed by laser scanning of bound phycoerythrin. These scan results are posted on HopGene ftp server and are freely accessible at 

### Expression Data Analysis

**Figure 8:**
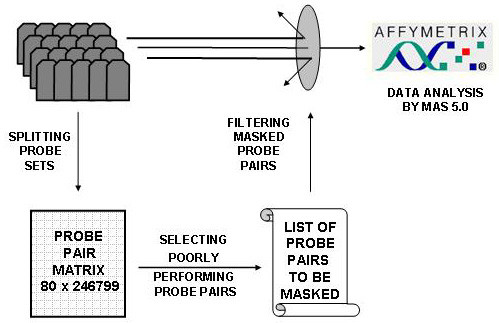
Schema of Affymetrix data analysis restricted to excellent performing probe pairs.

Data analysis was conducted using Affymetrix MicroArray Suit 5.0 (MAS 5.0) and GeneChip Operating Software (GCOS 1.1) (Affymetrix Inc., Santa Clara, CA). Signal intensity values for hybridization of human samples or heterohybridization of canine samples to HG-U133A (40 GeneChips for each specie), and hybridization of canine lung tissues to Affymetrix canine GeneChips (12 GeneChips) were generated using user-definable parameters set to Affymetrix default values and total chip fluorescence intensities scaled to 150. Each GeneChip was analyzed with and without application of custom generated probe masking files. The probe masking function is imbedded into MAS 5.0 and GCOS 1.1 and can be easy evoked and linked to a list of probes selected for masking. The overall schema of analysis of 40 human HG-U133A GeneChips, which were hybridized with canine mRNA is outlined in Figure 8.

### Creating "Perfect match"/"Mismatch" probe level matrix

Each HG-U133A GeneChip contains 22,215 (excluding internal controls) probe sets, each representing one target sequence. The majority of the probe sets (98 %), contain 11 probe pairs with each probe pair consisting of two 25-mer oligonucleotides. One of these oligonucleotides is a "perfect match" (PM) to a target sequence and another is a "mismatch" (MM) with an intentionally mutated middle nucleotide (Figure 1A). Therefore, each probe set contains 11 PM probes and 11 corresponding MM probes for calculation of total and nonspecific hybridization signals, respectively (reader is referred to Affymetrix website for detailed explanations, ). The MAS 5.0 program analyzes the differences in the amount of hybridized mRNA to each PM (total signal) and MM probes (background signal) across entire probe set, and computes the relative abundance of a transcript and its significance (*p *value). Transcripts with p < 0.04 are called "Present," with 0.04 ≤ p ≤ 0.06 called "Marginal," and with p > 0.06 called "Absent". The evaluation of the effects of our masking approach on the change in the transcript detection call was based solely on the Affymetrix transcript abundance classification without modification of the original algorithm, which makes employment of our masking files universal among Affymetrix users.

Expression profiles analyzed in this report were generated using total mRNA extracted from different parts of canine lungs exposed to 15 different experimental conditions. Signal intensities files (CEL files) generated by MAS 5.0 and GCOS 1.1 for HG-U133A and Canine GeneChip, respectively were imported into the R-based Bioconductor analytical package  and processed using *affy *library [23]. Intensity signals for each experimental group were corrected for background and normalized using default *mas5 *package settings, which closely simulate the MAS 5.0 algorithm. The raw intensities of PM and MM values for each of 246799 probes per chip (excluding internal Affymetrix controls) were extracted from *mas5 *generated probe level matrix [24] and converted into the flat text file with 80 by 246799 entries (Figure 8). The R-language scripts for extraction of PM and MM values are available upon request. Further manipulations and analyses of extracted data were conducted using HopGene designed algorithms written in Python 2.2. Statistical parts of the script employed the Python Statistical Module developed by NSG group 

### Correlation of the human-probe/canine-target similarity and hybridization signal

To evaluate relation between probe/target sequence similarity and hybridization signal all probe pairs were classified as efficient (PM-MM>20, the minimum practical signal value [25]) or inefficient (PM-MM≤20). This classification was applied to individual probe pairs throughout 40 canine/human hybridizations and 40 human/human hybridizations. Probe level analysis of human/human hybridization was aimed at identification of non-functional due to poor design or inappropriate hybridization conditions probe pairs (Figure 1B). These probes were used for identification of the net effect of canine/human genetic differences, that is probes that were inefficient in both human/human and canine/human hybridizations (Figure 4) were eliminated from further analysis. Target sequences (FASTA format) representing canine net effect probes (Figure 4) were retrieved from NetAffx  and queried against NCBI non-redundant (*nr*) database for their corresponding canine orthologues using MegaBLAST . Since the MegaBLAST batch query tool limits the number of submitting sequences, we divided this human dataset into 3 batches (~7000 sequences each) and used *Canis familiaris *term as the search restriction. BLASTN 2.2.8 [Jan-05-2004] analysis employed sequences form GenBank, EMBL, DDBJ, PDB databases, excluding EST, STS, GSS, or phase 0, 1 or 2 HTGS sequences and showed that 662 target sequences on the HG_U133A GeneChip had 1122 canine counterparts with >75% sequence homology. For manual evaluation 20 target sequences were randomly selected from the pool of sequences that contained probes efficient in less that 5% hybridizations (hybridized only to 0–2 chips, Figure 4). Since each target sequence contains 11 probes the total of 220 probes was evaluated and 22 probes that efficiently hybridized only to 0–2 GeneChips (inefficient group) and 31 probes that efficiently hybridized to 38–40 (95%) chips (efficient group) were aligned to their canine counterparts. Alignments of these 53 probes to their canine counterparts were generated using CLUSTALW  and nucleotide mismatches were counted and accordingly assigned to the "efficient" or "inefficient" group. Since mismatch distribution was heavily skewed towards small numbers of mismatches (1–2) the correlation between probe performance and target sequence homology (number of identified mismatches) was evaluated using nonparametric Mann-Whitney test with *p *< 0.05 used as a significance cut-off (Figure 4, insert).

### Creating probe pair masking files

Hybridization of individual probe pairs (246799total) to each GeneChip (40 total) was evaluated and inefficient probe pairs that produced hybridization signal below minimum practical signal value [25] (PM-MM≤20) were identified. The flat text file containing two columns was generated, with the first column containing probe set ID entry and the second column containing order numbers of probe pairs to be masked. The empty template masking file was generated using MAS 5.0 masking tool and subsequently populated with data from the flat text file. The first masking file was built from probe pairs that were inefficient in all 40 hybridizations. The second masking file contained probe pairs that were inefficient in ≥35 hybridizations. The subsequent masking files were generated with 5 chosen as an increment of inefficient hybridizations with the last masking file comprised of probe pairs that were inefficient in the only one hybridization. Resulting 9 masking files (40, 35, 30, 25, 20, 15, 10, 5, 1) were applied during MAS 5.0 analysis of canine and human mRNA hybridizations to the HG-U133A GeneChips. The highest percentages of the "Present" call (Figure 5) were generated around the point at which probes that were inefficient in ≥15 hybridizations (approximately 40%) were masked and was considered an optimum condition for a given dataset. In further discussions the percentages of the described above masking points were conveniently approximated.

### Evaluating effects of masking approach

To simplify the evaluation of our masking procedure, only probe-sets containing 11 probe pairs, which comprises ~98% of all HG_U133A probe-sets, were assessed. The remaining 2% of probe-sets served as internal controls (20 probe pairs per set) and probe sets built from 14–16 probe pairs were inherited from the HG_U95AVs2 GeneChip. Our masking probe pair list for 11-member probe-sets contained 156,174 probe pairs (~65% of total). All counting and poorly performing probe selecting protocols were scripted in Python 2.2  and are available upon request.

Concordance of the masking HG_U133A probes with their poor homology to the corresponding canine counterparts was evaluated using analysis of 5 human/canine alignments including one presented in Figure 2C and four randomly selected alignments from three representative pools of probe sets: probe sets where all probes were detectable, probe sets where none of the probes were detectable and probe sets represented by both detectable and non-detectable probes .

### Affymetrix Canine and HG_U133A GeneChip comparison

Identification of homologous to Affymetrix Canine GeneChip target sequences on human HG_U133A GeneChip was conducted using MegaBLAST. The Affymetrix Canine GeneChip's target sequences in FASTA format were retrieved from NetAffx, split into 12 batches (~2000 sequences each) to overcome MegaBLAST limitations and queried against *nr *database using *Homo sapiens *term for filtering. GeneBank accession numbers of human orthologues identified by MegaBLAST for canine chip targets were matched against those on HG-U133A GeneChip using Microsoft Access 2000. Detection calls for common orthologues between 40 cross-hybridized HG-U133A GeneChips and 12 same-species hybridized canine GeneChips were compared. The transcripts that were efficiently detected in ≥ 40% (described above optimum condition) of tested GeneChips were considered as expressed. Then detection of these expressed in canine lung tissues transcripts was compared with their corresponding detection in original and masked cross-hybridization of canine lung tissues to human HG-U133A GeneChip. Transcripts detected by Canine GeneChip but missed by HG-U133A cross-hybridization were "false negative" and those not detected by Canine GeneChip but shown by HG-U133A heterohybridization were "false positive".

## Authors' contributions

DNG originated the probe level analysis of cross-species hybridization, carried out the bioinformatics studies and drafted the manuscript. SFM led the human component of the project and conducted human sample selection and hybridization analysis. BAS led the canine component of the project, originated and designed canine ventilation model, provided canine samples for hybridization, and revised the manuscript. RAI participated in the design of the study, established Bioconductor database for new canine GeneChip and consulted on the statistical analysis. SQY optimized protocols for canine samples hybridization to human and canine GeneChips, and assured the quality of generated microarray data. JGNC conceived of the study, supervised and coordinated the project, revised and advised on the manuscript preparation. All authors read and approved the final manuscript.
